# Recent advances in understanding the mechanisms in skeletal muscle of interaction between exercise and frontline antihyperglycemic drugs

**DOI:** 10.14814/phy2.16093

**Published:** 2024-06-07

**Authors:** Sean A. Newsom, Matthew M. Robinson

**Affiliations:** ^1^ School of Exercise, Sport, and Health Sciences, College of Health Oregon State University Corvallis Oregon USA

**Keywords:** aerobic, glucagon‐like peptide 1 receptor agonist, metformin, mitochondria, resistance, sodium‐glucose cotransporter‐2 inhibitor

## Abstract

Regular exercise and antihyperglycemic drugs are front‐line treatments for type‐2 diabetes and related metabolic disorders. Leading drugs are metformin, sodium‐glucose cotransporter‐2 inhibitors, and glucagon‐like peptide 1 receptor agonists. Each class has strong individual efficacy to treat hyperglycemia, yet the combination with exercise can yield varied results, some of which include blunting of expected metabolic benefits. Skeletal muscle insulin resistance contributes to the development of type‐2 diabetes while improvements in skeletal muscle insulin signaling are among key adaptations to exercise training. The current review identifies recent advances into the mechanisms, with an emphasis on skeletal muscle, of the interaction between exercise and these common antihyperglycemic drugs. The review is written toward researchers and thus highlights specific gaps in knowledge and considerations for future study directions.

## 1 DRUGS AND AEROBIC EXERCISE AS FRONTLINE TREATMENT FOR TYPE‐2 DIABETES

Antihyperglycemic drugs and increased physical activity are frontline treatments for tens of millions of adults with type‐2 diabetes (T2D) and related metabolic disorders (American Diabetes Association Professional Practice C, 2024). Drugs and exercise can each impact skeletal muscle, the primary tissue for insulin‐stimulated glucose uptake (DeFronzo & Tripathy, 2009), yet there remain key gaps in knowledge regarding their combined effects. This review focuses on the interaction of drugs and exercise on mechanisms regulating skeletal muscle metabolism and glucose uptake. Pubmed was searched for metformin, sodium‐glucose cotransporter‐2 inhibitors (SGLT2i; −gliflozins) and glucagon‐like peptide 1 (GLP1) receptor agonists (−glutides) and exercise. Metformin has the longest history of use with more studies on mechanisms versus the newer classes of SGLT2i and GLP1 receptor agonists. This review emphasizes mechanistic studies in human and animal models published in the last 5 years.

## 2 METFORMIN

### 2.1 Primary clinical response and mechanisms of action

**FIGURE 1:**
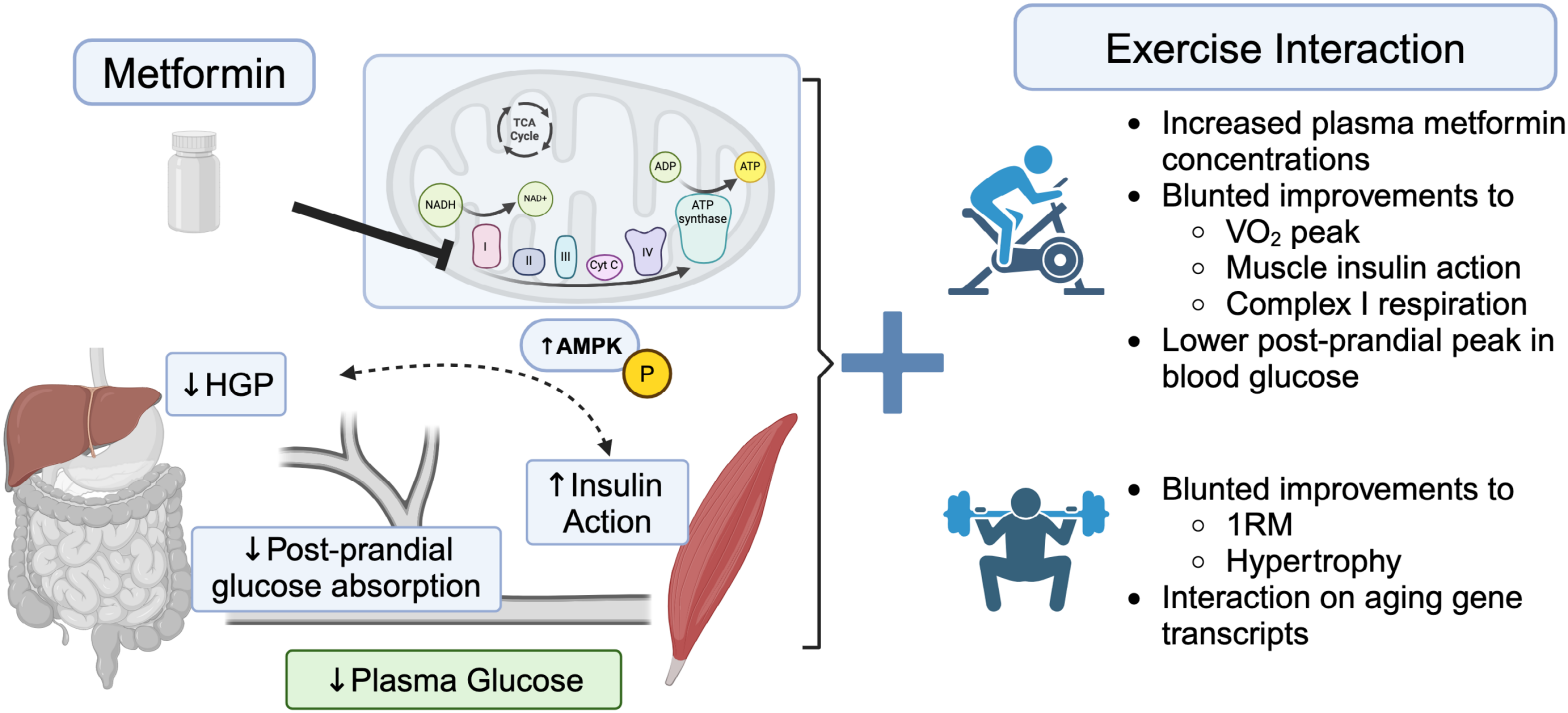
Metformin lowers glycemia through combined actions to lower hepatic glucose production (HGP) and possible complementary improvements to skeletal muscle insulin action. The combination of metformin with exercise can blunt several positive adaptations to aerobic and resistance training. Underlying mechanisms appear to be related to mitochondrial energetics. Image created with Biorender.com.

Metformin is the primary antihyperglycemic drug for T2D (Hughes et al., 2022) and is being considered in aging (Barzilai et al., 2016), autoimmune conditions (Ursini et al., 2018) and cancer (Lord & Harris, 2023). Metformin lowers fasting and postprandial glycemia (Figure 1). It decreases gluconeogenesis (Cusi et al., 1996; Stumvoll et al., 1995) via increasing ADP and NAD^+^ concentrations with AMPK‐dependent (Wang et al., 2019) and independent actions on glucose metabolism (see Rena et al., (2017) for detailed review). Changes to glucagon sensitivity and increased glucose production prevent hypoglycemia (Konopka et al., 2016). Metformin increases skeletal muscle insulin sensitivity (Eriksson et al., 2007; Konopka et al., 2016; Musi et al., 2002; Stumvoll et al., 1995), although results vary (Cusi et al., 1996).

Metformin can inhibit mitochondrial complex I (Drake et al., 2021), which in muscle is sufficient to increase muscle glucose uptake even independent of AMPK (Hou et al., 2018). The high concentrations used in model systems to inhibit complex I raise questions if such concentrations are achieved in‐vivo (Pavlovic et al., 2022; Wang et al., 2019). Drug concentrations depend on treatment duration. Short‐term treatment in humans with metformin (up to 3 days) was not sufficient to induce AMPK (Kristensen et al., 2019), but longer studies demonstrate metformin treatment inhibited gains in complex I after 12 weeks of moderate intensity aerobic training (Konopka et al., 2019). Metformin is also a weak cation at physiological pH, so the high mitochondrial membrane potential (negative inside) is predicted to accumulate metformin in the matrix at concentrations of 1000 times the extracellular medium (Owen et al., 2000). Longer treatments and pharmacodynamics could drive sufficient concentrations to inhibit mitochondrial complex I.

### 2.2 Metformin‐exercise interactions on glucose metabolism

Exercise may impact metformin concentrations. Using a one‐legged exercise model, Kristensen et al. reported that a single bout of cycling (40 min at about 80% peak workload) increased metformin concentrations in plasma and working muscle (Kristensen et al., 2019). Boule et al. reported plasma metformin concentrations in patients with T2D were greatest after they walked at intensities above ventilatory threshold (5 min) versus at intensities equal to or below their ventilatory threshold (15 min each) (Boule et al., 2011). The higher concentrations occurred alongside lower plasma glucose, free fatty acids and insulin concentrations after either the exercise or a standardized meal (Boule et al., 2011). The mechanisms driving metformin concentrations during exercise have yet to be identified and may include altered blood flow, renal excretion, etc.

The timing of metformin and exercise can impact glucose metabolism. A single exercise session (5 × 10 min walking at 60% maximum) at 30 min after a meal enhanced metformin's ability to lower the postprandial peak and 2‐h area under curve for glucose (Erickson et al., 2017). In patients with T2D, delaying exercise to 3 h after a meal raised postprandial glycemia compared with metformin alone (Myette‐Cote et al., 2016). Metformin concentrations were not reported, so it is not clear if greater concentrations after acute exercise drive lower postprandial glucose.

Some reports indicate metformin alone lowers postprandial glycemia without interaction with exercise. Pilmark et al. studied glucose kinetics in overweight and obese adults after a 3‐week lead‐in period with metformin (2000 mg daily) or placebo followed by 12 weeks of cycling training during metformin or placebo (Pilmark et al., 2021). Mixed meal testing with isotopic tracers revealed metformin lowered the rate of glucose appearance into blood from the meal, with no further change after exercise training.

Studies have reported negative interactions for metformin to blunt improvements to muscle insulin sensitivity following aerobic exercise. Randomized placebo control studies of adults with insulin resistance demonstrated that metformin consumption (2000 mg/day but not on testing days) blunted the increase in insulin sensitivity (compared with placebo) after either acute exercise or 12 weeks of aerobic training (Malin et al., 2012; Sharoff et al., 2010). Participants who had both impaired fasting blood glucose and glucose tolerance had more prominent blunting of exercise effects, suggesting that the degree of diabetes progression may influence the metformin‐exercise interaction. Viskochil et al. also reported that the combination of metformin and 12 weeks of aerobic training in patients with prediabetes blunted improvements in insulin sensitivity by ~25% compared with exercise alone (Viskochil et al., 2017). Such findings indicate a direct interaction of metformin on skeletal muscle adaptations during exercise training.

### 2.3 Metformin‐exercise interactions on skeletal muscle

Metformin's ability to inhibit complex I is a possible mechanism for interaction with aerobic training. Recent findings suggest that background mitochondrial activity correlates to metformin‐exercise response of mitochondria and insulin sensitivity. Konopka et al. studied older adults training 3 days per week for 45 min progressing to 85% of maximal heart rate (Konopka et al., 2019). Mitochondrial respiration declines with aging and improves with aerobic training, particularly at higher intensities (Robinson et al., 2017), yet Konopka et al. reported metformin treatment (1500–2000 mg daily) attenuated gains in *V*O_2_ peak, insulin sensitivity and complex I respiration (Konopka et al., 2019). There was a positive relationship between the change in complex I respiration and insulin sensitivity following aerobic training with placebo, but not with metformin. Baseline respiration correlated to training response so metformin's effects may depend on starting levels of respiration. Pilmark et al. did not report such hinderance in adults with glucose intolerance who trained for 12 weeks of 4 bouts of cycling per week for 45 min at average intensity of 64% maximal aerobic power. Aerobic training increased absolute *V*O_2_ peak by ~10% regardless of placebo or metformin (2000 mg daily) (Pilmark et al., 2021). Follow‐up studies indicated metformin treatment in adults with glucose intolerance did not hinder increases in citrate synthase activity or AMPK activity after acute exercise or 12 weeks of aerobic training (Pilmark et al., 2022). Training did not change mitochondrial respiration in either group, so it is not clear if metformin restricted complex I adaptations. A necessary advance in metformin‐exercise interaction is the influence of background respiration and mechanisms of drug interaction on mitochondrial metabolism.

Metformin may also impact adaptations to resistance training. In older adults without T2D, combining metformin with progressive resistance training, compared to resistance training alone, restricted gains in hypertrophy and maximal leg strength (+24% training vs. 15% combined) (Walton et al., 2019). Participants on statins had better improvement than those who only took metformin with training, suggesting combining statins plus metformin promoted gains in fiber cross sectional area (Long et al., 2022). Metformin has anti‐inflammatory effects (Cameron et al., 2016) that may impact muscle size and function. As evidence for a role on fiber size, metformin treatment prevented declines in fiber cross sectional area in older adults after 5 days of bed rest, a model of disuse atrophy (Petrocelli et al., 2023). Yet, Walton et al. reported no additional effects of metformin on increases in macrophage activation in muscle following resistance training (Walton et al., 2019). Beyond inflammation, the combination of metformin with training, versus training alone, induced a unique set of gene transcripts associated with aging (e.g., cell senescence, apoptosis, autophagy) and had high overlap with untrained younger adults (Kulkarni et al., 2020). Studies such as Targeting Aging with Metformin (TAME) will help identify efficacy of metformin on healthspan and expanding use in older adults (Barzilai et al., 2016).

### 2.4 Summary and gaps regarding metformin and exercise

**FIGURE 2:**
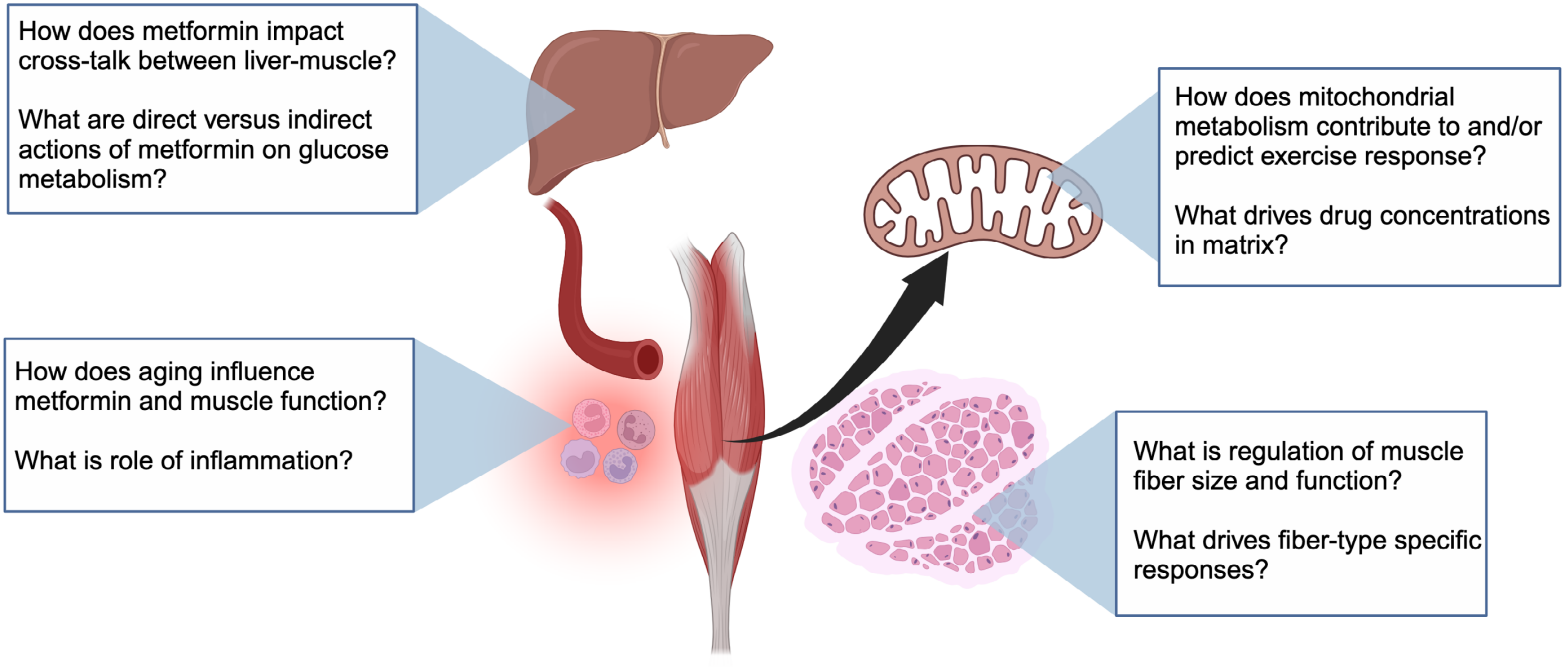
Future questions for metformin and exercise. Gaps include regulation of substrate metabolism across fiber types, separating resistance versus aerobic training adaptations, and cross‐talk with signals from other tissues. Image created with Biorender.com.

The high use of metformin to treat diabetes and expanding use to other conditions with impaired muscle function, such as aging, require a greater understanding of the interaction of metformin and exercise on skeletal muscle physiology (Figure 2). Combining metformin with aerobic training may blunt anticipated improvements in muscle insulin sensitivity and aerobic fitness, possibly through complex I activity. The mechanistic link remains to be made between metformin's actions on complex I and restricted gains in glucose metabolism or aerobic fitness. Identifying physiological indicators of responses may help identify patients who may benefit from treatment.

## 3 SODIUM‐GLUCOSE COTRANSPORTER‐2 INHIBITORS (SGLT2i)

### 3.1 Primary clinical response and mechanisms of action

**FIGURE 3:**
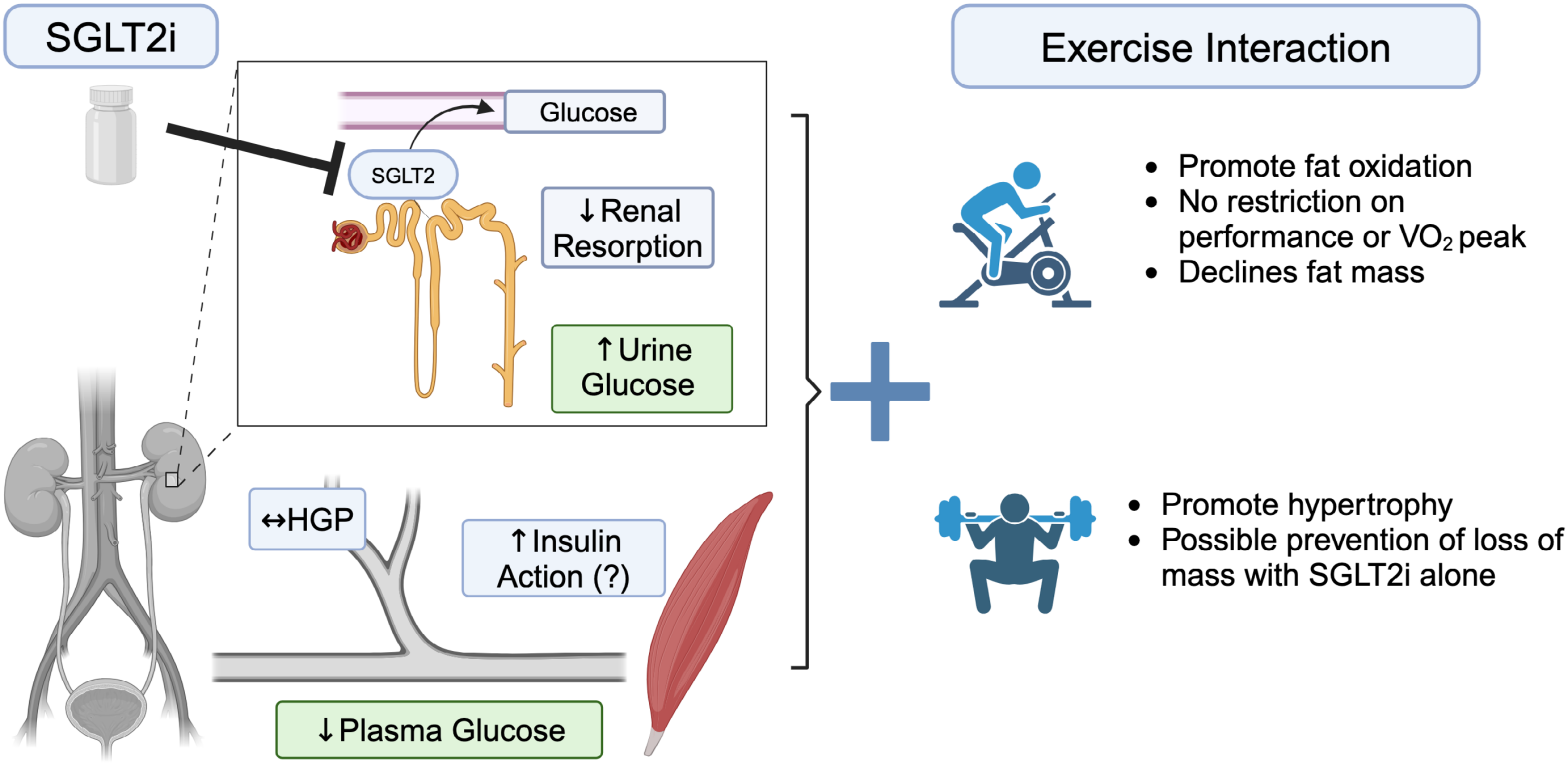
SGLT2i have primary actions to lower glucose resorption in kidneys that combine with off‐target actions on nonrenal tissues to lower plasma glucose. SGLT2i treatment during aerobic training can promote fat oxidation without limiting gains in performance. Resistance training may prevent loss of muscle mass with SGLT2i. Image created with Biorender.com.

SGLT2i received FDA approval in 2014 and are among the frontline medications for T2D. SGLT2i have high affinity to inhibit the 2 isoform of SGLT proteins within the proximal renal tubule, thereby lowering glucose reabsorption (Figure 3). Patients excrete upwards of 100 g of glucose per day to lower hyperglycemia (Ferrannini et al., 2014) while compensatory hepatic glucose output helps prevent hypoglycemia (Ferrannini et al., 2016).

Growing evidence indicates SGLT2i have off‐target actions on skeletal muscle (O'Brien et al., 2017), liver (Nakano et al., 2020), cardiac (Wang et al., 2020) and endothelial cells (Zugner et al., 2022). SGLT2 protein is expressed predominantly in kidney so a critical question is how does the drug work in skeletal muscle. Possible off‐target mechanisms are that SGLT2i treatment can lower complex I respiration (Hawley et al., 2016; Villani et al., 2016). Being a newer class of drugs, there is much to be understood for potential interactions of SGLT2i with exercise. Studies in humans have focused on how SGLT2i improve the cardiovascular response to exercise (Herring et al., 2023; Kayano et al., 2020) so there are only a few studies of SGLT2i and exercise on skeletal muscle.

### 3.2 SGLT2i‐exercise interactions on glucose metabolism

While SGLT2i and aerobic exercise can each improve glycemia, their combination does not appear to be additive on glucose metabolism. Newman et al. studied the interaction of dapagliflozin treatment and 12 weeks of cycling up to four times per week, 70%–80% of heart rate reserve in adults with obesity and found that training alone improved whole‐body insulin sensitivity (calculated via Matsuda index based on glucose and insulin response to a mixed meal), which was not seen after drug plus exercise (Newman et al., 2019). Dapagliflozin did not restrict the main positive effects of exercise on performance outcomes. Both placebo and dapagliflozin treatment resulted in lower heart rate and greater fat oxidation during exercise and improved *V*O_2_ peak after training (Newman et al., 2019). Fasting glucose was higher in the SGLT2i treatment group compared with training only, though both groups were normoglycemic (~82 vs. 73 mg/dL, respectively). In a rat model of T2D (streptozotocin treatment combined with high‐fat feeding), Linden et al. reported co‐treatment of dapagliflozin and exercise, compared with drug or exercise alone, resulted in a lower area under the curve for blood glucose and insulin response to a glucose tolerance test (Linden et al., 2019). The improved glucose tolerance occurred without changes to resting muscle glycogen concentrations (Linden et al., 2019). Overall, the combination of SGLT2i does not appear to be additive on improvements to glucose metabolism.

### 3.3 SGLT2i‐exercise interactions on skeletal muscle

SGLT2i can lead to loss of muscle mass in patients with type 2 diabetes (Zhang et al., 2023), creating a need to understand if exercise, particularly resistance, protects against loss of muscle mass. Bouchi et al. reported declines of 0.5 kg in fat free mass following 24 weeks of dapagliflozin (up to 10 mg daily) in patients with T2D who consumed dapagliflozin alone or in combination with at‐home resistance training (Bouchi et al., 2021). Adherence to the at‐home protocol is an important consideration because, while not showing interaction of drug and exercise on fat‐free mass, participants with adherence above 80% also decreased fat mass by nearly 2.5 kg while those with <60% lost ~1.5 kg. Future studies need to include supervised training to determine resistance training effects on muscle mass.

Loss of mass may be specific to fiber type. Rodent studies minimize adherence considerations and indicate training, particularly resistance, blunted loss of fast twitch fibers during SGLT2i treatment. Six weeks of dapagliflozin treatment in streptozotocin‐induced diabetic rats lowered mass for some muscles (extensor digitorum longus, EDL) but not others (soleus or gastrocnemius), indicating that loss of mass affects muscles with a greater proportion of type II fibers (Yang et al., 2023). Compared to dapagliflozin only treatment, rats that also had ladder training had greater mass and fiber cross sectional area for EDL. Soleus mass was similar across treatments and exercise groups. The overall gains in mass or fiber area after aerobic training did not reach statistical significance, suggesting that resistance training was more effective to promote hypertrophy during SGLT2i treatment, particularly with sufficient feeding.

Negative energy balance contributes to muscle loss, so compensatory increases in food intake may attenuate muscle loss with SGLT2i. For example, in a nondiabetic mouse model, mice treated with canagliflozin ate more food and had similar muscle mass to untreated mice. Yet, when food intake was matched between groups, the canagliflozin‐treated mice had lower muscle mass, particularly in fast twitch muscles, than un‐treated mice (Otsuka et al., 2022). Thus, food intake is an important variable on SGLT2i changes to muscle mass and body weight, while implications on impacting performance for weight‐bearing exercise. For example, compared to treadmill training alone, rats that trained with dapagliflozin treatment had lower gains in body weight and fat mass alongside longer running distance at 75% *V*O_2_ peak (Linden et al., 2019). Energy balance must be accounted for as a variable on muscle mass and impact on exercise performance.

In addition to net loss of energy, SGLT2i, because of glucose excretion, shifts whole‐body metabolism toward fat oxidation (Ferrannini et al., 2016). The impact on muscle lipid metabolism is less clear and does not appear to be simply a loss of intramuscular lipids or higher lipid oxidation. Op den Kamp et al. reported that 5 weeks of dapagliflozin, in adults with T2D, increased muscle lipid abundance and droplet size along with higher concentrations of acylcarnitines (Op den Kamp et al., 2022). Mitochondrial respiration for either complex I, II or lipid substrates were not different after SGLT2i treatment. They next investigated muscle and plasma metabolites (measured by ^1^H‐magnetic resonance spectroscopy) after a single bout of 30 min of cycling, which lowered plasma lactate but not muscle acylcarnitines (Op den Kamp et al., 2022). The composition and cellular location of intramuscular lipids are associated with insulin action (Kahn et al., 2021). Key remaining questions include how does SGLT2i impact muscle lipid turnover and what is the interaction with aerobic training.

The combination of canagliflozin treatment during 6 weeks of voluntary wheel running in a mouse model of hyperglycemia without obesity improved gains in *V*O_2_ peak and running distance than exercise alone, indicating that SGLT2i may improve exercise performance, in part, through lowering hyperglycemia (MacDonald et al., 2022). High‐fat fed mice had less voluntary wheel running when treated with canagliflozin (5 vs. 7 km daily in untreated mice), yet despite such difference in exercise habits, the mice had similar fasting insulin concentrations and lowered glucose response during glucose tolerance test (Tanaka et al., 2020). Tanaka et al. further reported that versus canagliflozin alone, combining wheel running with canagliflozin treatment shifted whole‐body substrate oxidation toward lipids alongside changes in gene profiles in gastrocnemius muscle toward lipid oxidation and angiogenesis (Tanaka et al., 2020).

Several lines of evidence indicate SGLT2i have off‐target effects to inhibit mitochondrial respiration, particularly at complex I. Inhibition of complex I occurs at in‐vivo concentrations across several cell types including renal cell cultures (Secker et al., 2018), prostate and lung cancer cells (Villani et al., 2016), and liver tissues from mice treated with canagliflozin (Hawley et al., 2016). Downstream of complex I, the drug effects appear dependent on AMPK activation which implicates a mechanism of AMP buildup and activation of AMPK (Hawley et al., 2016). Molecular docking studies indicate SGLT2i may bind to AMPK (Smith, 2001; Song et al., 2022), providing a possible direct way to activate AMPK beyond actions on complex I. Overexpressing complex I subunit (ND1) abolished effects of canagliflozin on cell proliferation (Villani et al., 2016), which implicates the inhibition of complex I as an off‐target mechanisms of SGLT2i. The inhibition appears to be dose dependent and vary between drugs, with canagliflozin having more potent inhibition than dapagliflozin in liver cell cultures (Hawley et al., 2016). It needs to be determined if inhibition of respiration is different between SGLT2i formulations.

Longer term effects of SGLT2i on skeletal muscle mitochondria are not clear. Op den Kamp et al. reported no difference in complex I respiration of muscle fibers after 5 weeks of placebo or dapagliflozin treatment in patients with T2D (Op den Kamp et al., 2022), or after 2 weeks in adults with prediabetes (Veelen et al., 2022). Hawley et al. did not find activation of AMPK in skeletal muscle from treated mice (Hawley et al., 2016). Measuring complex I activity in skeletal muscle requires tissue excision and processing such that drug diffusion kinetics may affect ex‐vivo measures of complex I versus in‐vivo effects of SGLT2i. SGLT2i are weakly charged at in‐vivo pH so could freely diffuse across membranes. Evidence also indicates that SGLT2i may be transported into nonrenal tissues. Blocking activity of organic anion transporter three raiseblood concentrations of empagliflozin (Fu et al., 2018). Determining in‐vivo actions of SGLT2i on mitochondria will require understanding of the transport/diffusion of these drugs across membranes and ability of SGLT2i to access and bind mitochondrial proteins.

### 3.4 Summary and gaps regarding SGLT2i and exercise

**FIGURE 4:**
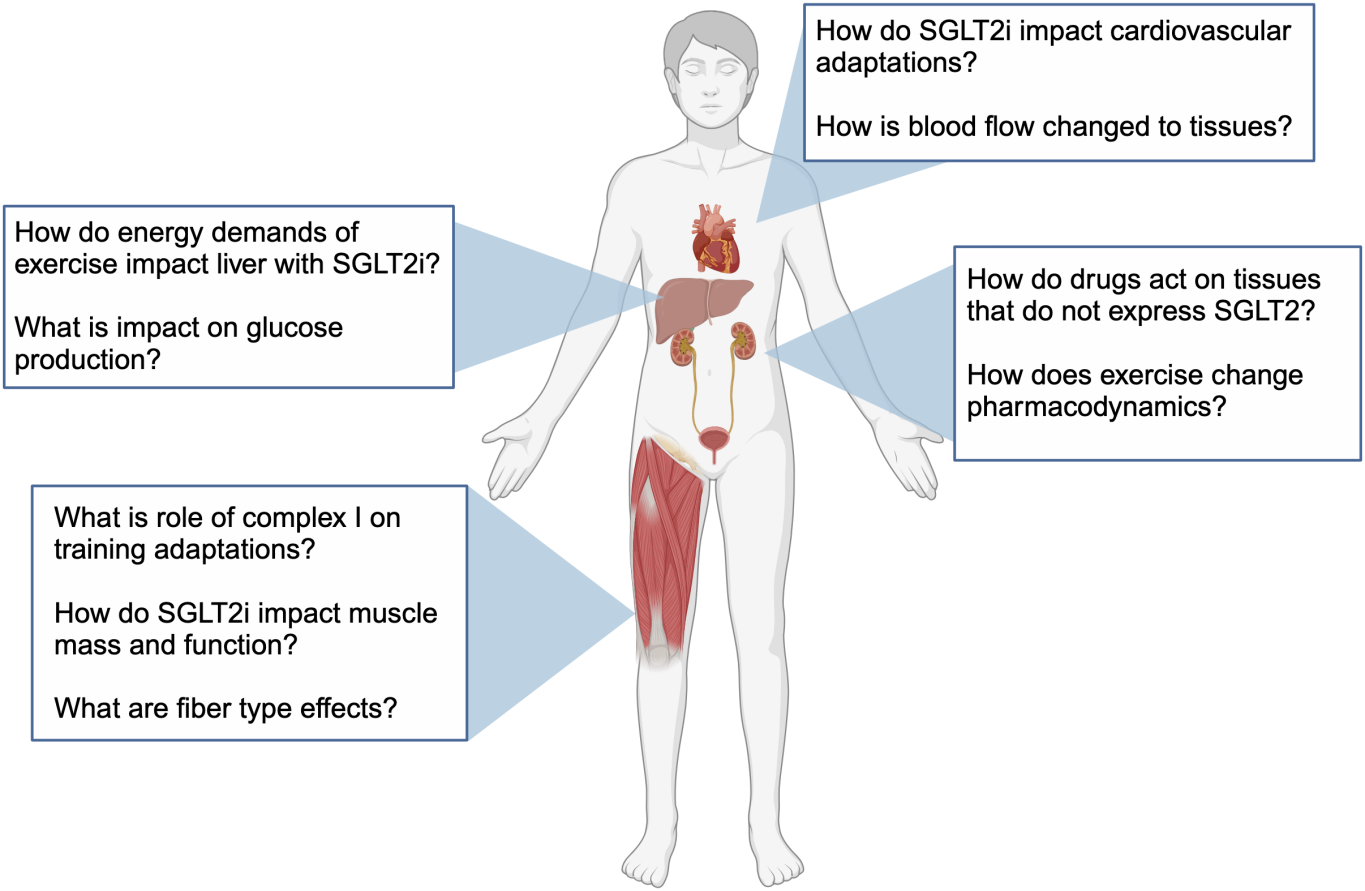
Future questions for SGLT2i and exercise. Fundamental questions are rooted in how SGLT2i have off‐target effects in muscle and other tissues that do not express SGLT2 protein. Image created with Biorender.com.

The use of SGLT2i is expanding for treatment of T2D and related metabolic conditions. Current studies do not indicate an additive effect of SGLT2i and exercise on improvements to glucose metabolism. A key fundamental gap is identifying mechanisms by which SGLT2i have off target effects on nonrenal tissues including transport of drug across membranes (Figure 4). The impact of SGLT2i on mitochondrial adaptations to exercise are not known despite several lines of evidence showing SGLT2i can lower complex I respiration. Fiber type differences between aerobic and resistance training must be determined including susceptibility to atrophy and impact on contractile and metabolic function. Knowing off‐target actions is foundational to understanding how SGLT2i promote beneficial cardiovascular and metabolic outcomes.

## 4 GLP1 RECEPTOR AGONIST

### 4.1 Primary clinical response and mechanisms of action

**FIGURE 5:**
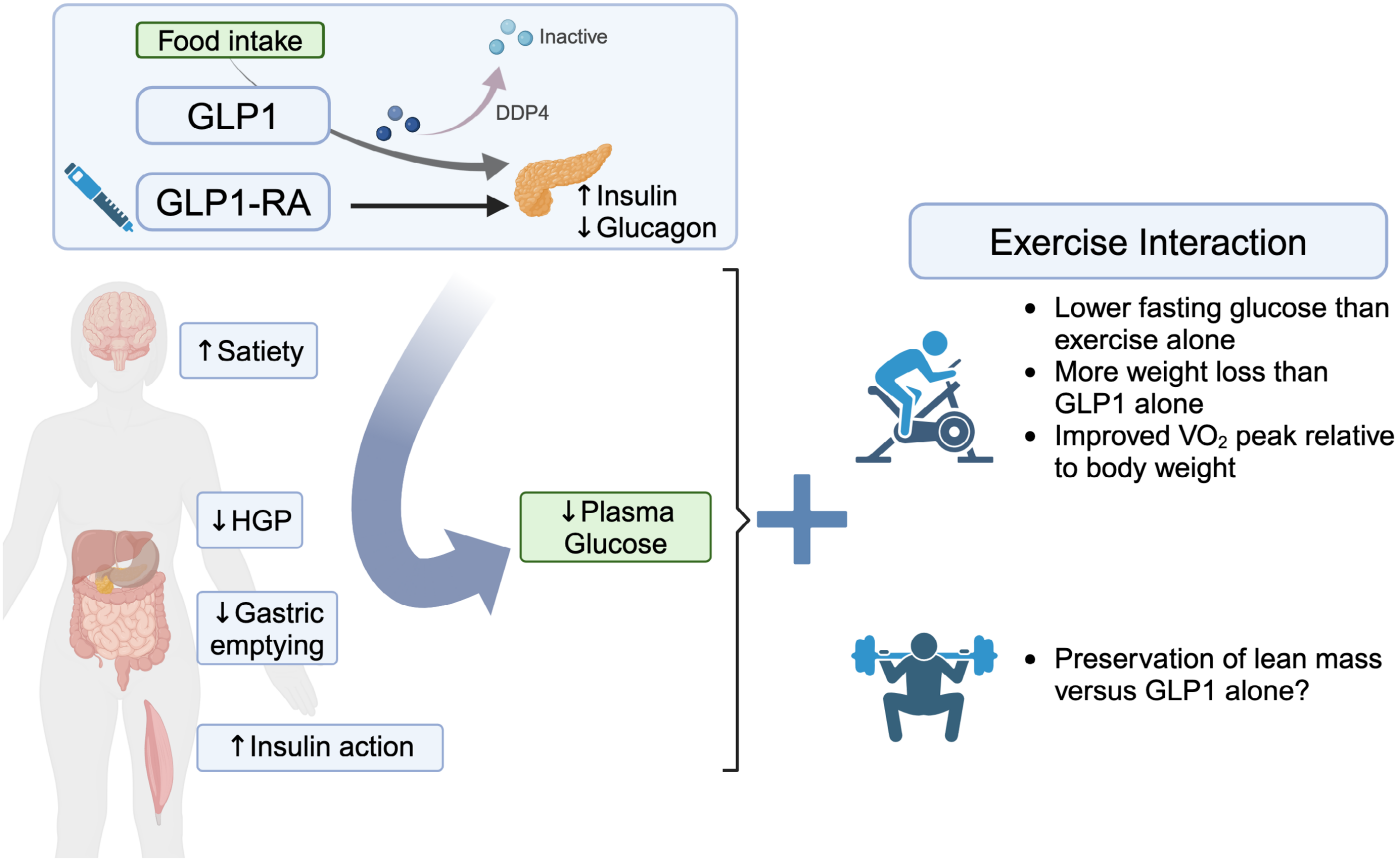
GLP1 receptor agonists provide longer term GLP1 signaling and incretin effect, with downstream impact on lowering blood glucose and weight loss. The combination with exercise can further promote weight loss while maintaining muscle mass. Image created with Biorender.com.

GLP1 is secreted from the small intestine and stimulates insulin release to increases in blood glucose (Figure 5). GLP1 is inactivated by dipeptidyl peptidase 4 (DPP‐4) within 1–2 min, so the structural design of GLP1‐ receptor agonists, “−glutides”, avoids degradation by DPP‐4 to promote GLP1 signaling. Glutides are often combined with insulin sensitizing drugs to lower hyperglycemia. Demand for GLP1 receptor agonists has increased since 2021 when the FDA approved semaglutide for weight loss treatment. Exercise is recommended with GLP1‐receptor agonist treatment so, being a newer drug class, the growing use creates an urgent need to understand drug‐exercise interaction.

Changes in blood flow are an indirect mechanism by which GLP1 can impact muscle through influencing nutrient and hormone delivery. GLP1 signaling can improve diastolic pressure in sedentary adults with T2D (Scalzo et al., 2019). Such improvements occurred without improvement to cardiorespiratory performance or mitochondrial metabolism (measured via magnetic resonance spectroscopy). In a rat model of impaired myocardial function, which induces pronounced exercise impairment, promoting GLP1 signaling via a DPP4 inhibitor improved time to exhaustion and *V*O_2_ peak alongside increases in respiration and abundance of mitochondrial complexes I and II (Takada et al., 2016). Improvements to blood flow and vascular adaptations may promote exercise adaptations (discussed below).

In addition to blood flow, GLP1 receptor agonists have direct actions on pathways regulating muscle viability and function, such as autophagy which is needed to be activated to promote muscle mass (Masiero et al., 2009) and glucose adaptations during exercise training (He et al., 2012). Liraglutide prevented the loss of cell viability and increased autophagy markers during palmitate treatment in L6 cells (Tian et al., 2023). The direct actions of GLP1 on muscle combined with cardiovascular control provide several potential mechanisms by which GLP1 receptor agonists may interact with exercise training.

### 4.2 GLP1‐exercise interactions on glucose metabolism

Clinical trials indicate a positive interaction of exercise and GLP1 agonists on glucose concentrations. The combination of liraglutide with 16 weeks of combined exercise training (60 min per day for 2 days aerobic and 1 day resistance exercises per week) lowered fasting glucose more so than exercise alone in adults with T2D (−3.4 vs. −0.3 mM), resulting in more patients achieving HbA1c target ranges (−2.0 vs. −0.3 decrease in % HbA1c) (Mensberg et al., 2017). Ingersen et al. tested β‐cell responsiveness, using glucose tolerance testing and insulin clamp, in patients with T2D after semaglutide alone or combination with aerobic training (12 weeks of cycling three times week for 45 min at 75% heart reserve). The combination of drug and exercise improved β‐cell sensitivity to changing glucose more so than drug alone, indicating positive interaction on insulin secretion to improved glycemia with training (Ingersen et al., 2023). The mechanisms need to be identified by which GLP1 receptor agonists improve glucose metabolism during aerobic training, including separating direct actions on muscle versus indirect such as blood flow.

GLP1 receptor agonists may promote improvements to weight loss and aerobic fitness with exercise training. Lundgren et al. studied liraglutide and exercise in patients who had already lost over 5% of body weight (~13 kg) and found that combining exercise with drug treatment promoted additional loss of body weight, mostly fat mass, of ~5.4 kg more than exercise alone (Lundgren et al., 2021). Weight loss contributed to the drug‐exercise combination improving *V*O_2_ peak by ~5 mL/kg/min while exercise or liraglutide alone improved *V*O_2_ peak ~3 and ~1 mL/kg/min, respectively. The combined effects of drug and exercise on weight loss appear driven mostly by increased satiety and lower food intake, with less impact on energy expenditure (Blundell et al., 2017; Jensen et al., 2022).

### 4.3 GLP1‐exercise interactions on skeletal muscle

Studies report a positive interaction that aerobic exercise can prevent loss of mass during treatment with GLP‐receptor agonist. In their study testing β‐cell sensitivity in patients with T2D discussed above, Ingersen et al. reported that semaglutide treatment alone for 20 weeks decreased both fat and lean mass (~5 and 2 kg loss) then there were no further declines when patients combined treatment with cycling (Ingersen et al., 2023). In a 16 weeks study of weight loss in obesity, combining liraglutide to a diet and exercise program that aligned with physical activity recommendations (achieving 140 min moderate aerobic plus 1–2 days/week resistance training) augmented the loss of fat free mass (−2.3 kg vs. −1.5 kg, p = 0.06), indicating that the exercise recommendations were not sufficient to prevent loss of muscle mass (Grannell et al., 2021). Parameters such as intensity and mode need to be tested for preventing loss of fat free mass.

Studies on GLP1 receptor agonists have focused on incretin and satiety effects, so less is known regarding GLP1 signaling mechanisms on skeletal muscle adaptations to exercise. Activation of GLP1 signaling in skeletal muscle in mice (via either overexpression or injection of exendin‐4, a GLP1 receptor agonist) induced glycogen storage and increased time to exhaustion during an acute swim test (Wu et al., 2022). Further, treating C2C12 myotubes with exendin‐4 stimulated mtDNA and respiration via AMPK‐dependent mechanisms (Wu et al., 2022). These studies demonstrate GLP‐1 has direct actions on muscle.

Actions of GLP1 on the cardiovascular system may have indirect effects on muscle and exercise performance. Cardiac and vascular tissues have also been shown to require GLP1 signaling for exercise adaptations. For example, in a Goto‐Kakizaki rat model of insulin resistance, increasing GLP1 signaling with a DPP4 inhibitor during 3 weeks of treadmill training increased running time to exhaustion and markers of mitochondrial protein abundance in the aorta (Keller et al., 2015). An opposite approach to block GLP1 signaling in rats, using the GLP1 receptor antagonist, exendin 9–39 via subcutaneous osmotic pump, blunted aerobic capacity and several cardiac adaptations, including carotid artery strain and elasticity after 3 weeks of treadmill training (Scalzo et al., 2018). So, both agonist and antagonist approaches reveal GLP1 regulates cardiovascular adaptations and improves exercise performance. Changes in blood flow will have downstream effects on muscle performance, such as changes in fuel and oxygen delivery, and regulation of muscle mass, such as insulin and amino acids. Future studies will need to separate the direct actions of GLP1 on muscle versus indirect through the cardiovascular system, on muscle performance and adaptations.

### 4.4 Summary and gaps regarding GLP1 receptor agonists

**FIGURE 6:**
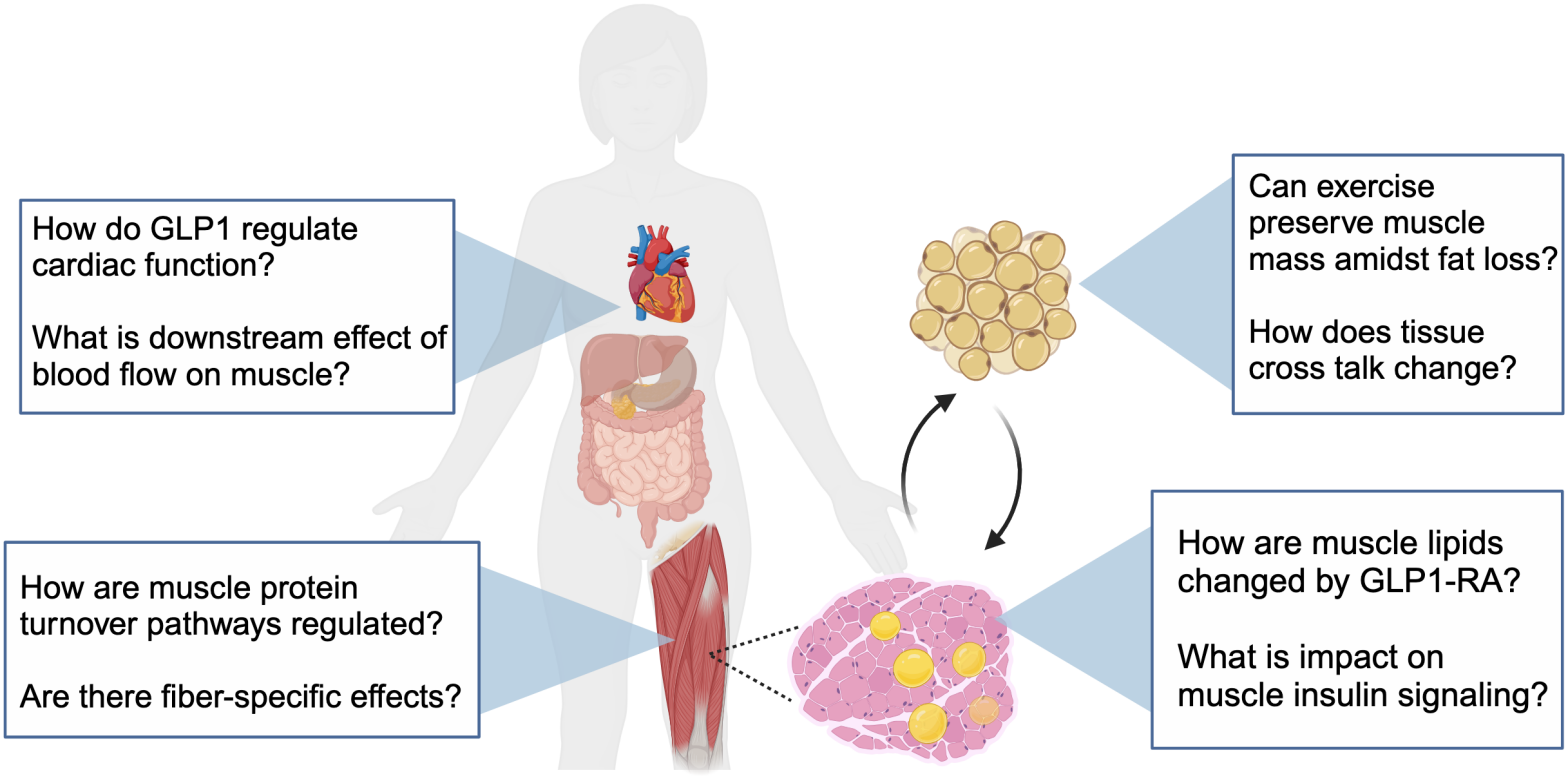
Future questions for GLP1 receptor agonists and exercise. Gaps include separating direct drug actions on skeletal muscle, including regulation of mass and function, versus indirect effects through cardiovascular regulation or decreased fat mass. Image created with Biorender.com.

The increasing use of GLP1 receptor agonists alongside exercise recommendations reveals several gaps regarding the impact on skeletal muscle beyond glycemic control (Figure 6). There is urgent need to identify if and how exercise could preserve skeletal muscle mass and function during weight loss. Future directions on muscle protein turnover need to determine the interplay between hyperinsulinemia on suppressing degradation versus the direct activation of autophagy by GLP1 receptor agonists. The role for GLP1 signaling to regulate cardiac function and vascular response (Jorgensen et al., 2017) raises important considerations for the interaction of exercise and GLP1 signaling direct on heart and vasculature, then downstream on nutrient delivery to skeletal muscle.

## 5 EMERGING CONCEPTS

Antihyperglycemic drugs and physical exercise will remain frontline treatments for T2D and related metabolic disorders with goals to improve glucose metabolism and physical function. Metformin treatment has led to heterogenous responses to exercise regarding improvements to insulin action or hypertrophy that may be related to mitochondrial energetics or aging‐related gene pathways. SGLT2i treatments have off‐target actions in nonrenal tissues that must be further explored including impact of drug on mitochondrial energetics in muscle and liver during exercise. The expanded use of GLP1 agonists for weight loss has revealed needs to better understand GLP1 signaling on muscle mass, bioenergetics and cardiovascular responses to exercise. Addressing these and other gaps regarding the interactions between antihyperglycemic drugs and physical exercise will enable refined intervention strategies that maximize their therapeutic potential.

## ETHICS STATEMENT

None.
